# Retraction: The Survival Benefit of Liver Transplantation for Hepatocellular Carcinoma Patients with Hepatitis B Virus Infection and Cirrhosis

**DOI:** 10.1371/journal.pone.0222176

**Published:** 2019-08-30

**Authors:** The PLOS ONE Editors

Concerns have been raised that the transplants performed in the local context at the time of procedures reported in this article [1] may have involved organs/tissues procured from prisoners [2].

Details as to the donor sources and methods of obtaining informed consent from donors were not reported in [1], and the authors did not clarify these issues or the cause(s) of donor death in response to journal inquiries. International ethics standards call for transparency in organ donor and transplantation programs and clear informed consent procedures including considerations to ensure that donors are not subject to coercion.

In addition, the article states that this study was “performed incompliance with principles of the Helsinki Declaration, and institutional guidelines” [1] but does not report whether this study received approval from a research ethics committee. The authors did not reply to post-publication queries from the journal asking for information and documentation about ethics approval for this study.

In addition, the authors did not respond to inquiries about the availability of underlying data supporting this study.

Owing to unresolved questions about whether this study had ethics approval, the lack of documentation to clarify this point, insufficient reporting, unresolved concerns around the source of transplanted organs and whether they included organs from prisoners, and in compliance with international ethical standards for organ/tissue donation and transplantation, the *PLOS ONE* Editors retract this article.

The authors did not respond to the retraction decision.
